# Adsorption isotherms in roasted specialty coffee (*Coffea arabica* L.): Dataset and statistical tools for optimizing storage conditions and enhancing shelf life

**DOI:** 10.1016/j.dib.2024.111247

**Published:** 2024-12-19

**Authors:** Gentil A. Collazos-Escobar, Andrés F. Bahamón-Monje, Nelson Gutiérrez-Guzmán

**Affiliations:** a Centro Surcolombiano de Investigación en Café (CESURCAFÉ), Departamento de Ingeniería Agrícola, Universidad Surcolombiana, Neiva-Huila 410001, Colombia; b Grupo de Análisis y Simulación de Procesos Agroalimentarios (ASPA), Instituto Universitario de Ingeniería de Alimentos–FoodUPV, Universitat Politècnica de València, Camí de Vera s/n, Edificio 3F, València 46022, España

**Keywords:** Hygroscopicity, Water adsorption properties, Thermodynamic properties, Moisture stability, Storage optimization, Quality improvement

## Abstract

This work presents a comprehensive dataset of adsorption isotherms and infrared spectral data for roasted specialty coffee (*Coffea arabica* L.). The dataset includes adsorption isotherms for whole roasted beans and ground coffee at medium (850 µm) and fine (600 µm) particle sizes. Adsorption isotherms were experimentally determined using the Dynamic Dewpoint Isotherm (DDI) method, considering a water activity range from 0.1 to 0.9 and temperatures of 25, 35, and 45 °C. Attenuated Total Reflectance-Fourier Transform Infrared (ATR-FTIR) spectroscopy was used to characterize the coffee samples spectrally. The dataset also provides two calibrated sorption models: Peleg and Double Logarithm Polynomial (DLP), which are highly suitable for practical applications, allowing users to predict the equilibrium moisture content of coffee as a function of water activity, temperature, and coffee type (whole beans or ground coffee). These tools offer valuable insights for researchers, coffee producers, and decision-makers in computing critical parameters related to roasted specialty coffee's shelf life, determining optimal packaging materials, and understanding the hygroscopic properties of coffee. The calibrated models could be used to optimize coffee storage processes and improve the understanding of water sorption behavior in different particle sizes. The dataset compiles the experimental adsorption isotherms and infrared spectra in Excel sheets according to the experimental conditions and replicates. Furthermore, this dataset includes different MATLAB® R2023a (The MathWorks Inc., Natick, MA, USA) files where the models have been calibrated with the experimental adsorption isotherms using the “Curve Fitting” tool and are available for their use in the coffee industry.

Specifications Table

**Table:** 

Subject	Food engineering
Specific subject area	Food technology, Food engineering, Food Science
Type of data	Excel files (Roasting curves of specialty coffee, Initial moisture content and water activity, Infrared spectra of specialty coffee, Experimental water adsorption isotherms). Figure (Roasting curves of specialty coffee, water adsorption isotherms and infrared spectral data, statistical fitting of adsorption isotherms using Peleg and DLP models, procedure on computer modeling of adsorption isotherms in roasted specialty coffee and experimental procedure for dataset acquisition and mathematical modeling). MATLAB files (Load data and open Curve fitting tool implementing both Peleg and DLP models).
Data collection	Water adsorption isotherms (Dynamic Dewpoint Isotherm analysis, DDI), infrared spectra (Attenuated Total Reflectance-Fourier Transform Infrared, ATR-FTIR).
Data source location	The experimental dataset described in this work has been obtained in the Centro Surcolombiano de Investigación en Café (CESURCAFÉ) from the Universidad Surcolombiana, Neiva-Huila, Colombia.
Data accessibility	Repository name: Mendeley Data Data identification number: 10.17632/6dnw4dymkp.1 Direct URL to data: https://data.mendeley.com/datasets/6dnw4dymkp/1
Related research article	G. A. Collazos-Escobar, N. Gutiérrez-Guzmán, H. A. Váquiro-Herrera, J. Bon, J V. García-Pérez, Thermodynamic analysis and modeling of water vapor adsorption isotherms of roasted specialty coffee (Coffee arabica L. cv. Colombia), LWT. 160 (2022) 113,335.

## 1 Value of the Data

• This dataset provides comprehensive adsorption isotherms for roasted coffee beans and ground coffee at various particle sizes, along with the infrared spectra of roasted coffee. These data are critical for understanding how roasting and particle size influence the moisture adsorption properties of coffee, which are essential for ensuring storage stability and shelf life. Additionally, the infrared spectra provide insights into the chemical composition of roasted specialty coffee, thereby offering insights into the functional groups and compounds that influence moisture adsorption and contribute to sensory characteristics.
• The dataset offers essential tools for optimizing storage processes, allowing predictions of equilibrium moisture content based on water activity, temperature, and coffee type. This is critical for preserving the sensory qualities of roasted coffee, such as flavor and aroma, by preventing moisture-induced deterioration.
• The dataset comprises two calibrated sorption models (Peleg and DLP), which facilitate the estimation of equilibrium moisture content. The included MATLAB® files, which contain compiled models, can be immediately applied in real-time applications within the coffee industry. These tools are of significant value to decision-makers seeking to implement precise, data-driven strategies for the management of coffee storage and the maintenance of quality over time. Moreover, researchers can utilize this data to calculate crucial parameters such as monolayer moisture content, adsorption surface area, and thermodynamic properties (Gibbs free energy, adsorption enthalpy, and entropy), thereby enhancing predictions of coffee behavior under different storage conditions.
• The dataset enables real-time monitoring of moisture content changes during the storage of specialty coffee. It also offers tools to assess the hygroscopic properties of coffee stored in different forms: whole beans, medium-sized particles, and fine-ground coffee, ensuring consistent quality across the various forms in which coffee is demanded.
• This dataset supports the calibration of chemometric and machine learning models, allowing for deeper analysis of how water activity, temperature, and particle size affect the moisture content and stability of roasted coffee.
• This dataset delivers essential knowledge and tools for researchers, coffee producers, and industry stakeholders, aiding in the optimization of storage conditions, packaging solutions, and quality control practices for roasted specialty coffee.

## 2 Background

In recent years, there has been a notable increase in global demand for specialty coffee, particularly *Coffea arabica* L., due to its distinctive flavor and aroma [1]. As quality becomes the primary factor driving market differentiation, the roasting techniques and storage conditions employed have become critically important in preserving the sensory attributes that define high-value coffee [2]. It is a well-established fact that roasted coffee is highly hygroscopic [3], meaning that it readily adsorbs moisture from its environment. This process can have a detrimental effect on flavor and significantly reduce the shelf life of the coffee. It is of utmost importance to ensure that storage conditions, particularly water activity and temperature [4], are properly managed in order to maintain the integrity of both roasted beans and ground coffee. The moisture uptake of coffee is influenced by a number of factors, including particle size, temperature, and relative humidity. It has been observed that ground coffee exhibits a greater capacity for moisture absorption than whole beans [5]. Adsorption isotherms offer significant insights into the water sorption behavior of roasted coffee, facilitating the prediction of equilibrium moisture content across diverse conditions [6]. Infrared spectroscopy provides further insight into the chemical changes that occur in coffee during storage [7]. Nevertheless, there is a lack of research examining the impact of temperature on roasted coffee's water adsorption behavior. This study addresses this gap by offering a comprehensive dataset of adsorption isotherms and infrared spectral data for roasted specialty coffee, providing researchers and industry professionals with the mathematical tools to optimize storage practices and maintain coffee quality.

## 3 Data Description

**Fig. 1:**
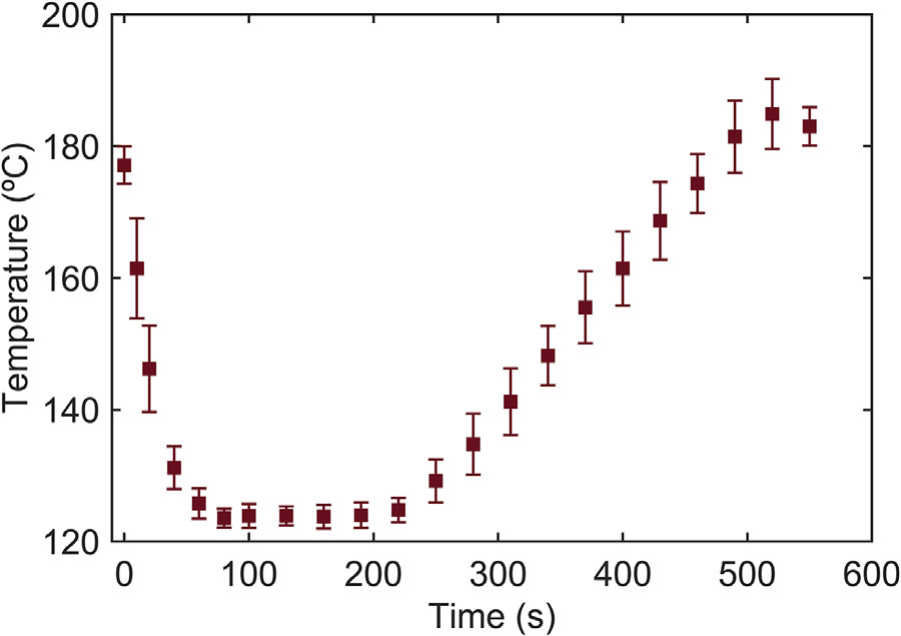
Coffee roasting curve illustrating the relationship between the temperature program and processing time for achieving a medium roast grade.

**Fig. 2:**
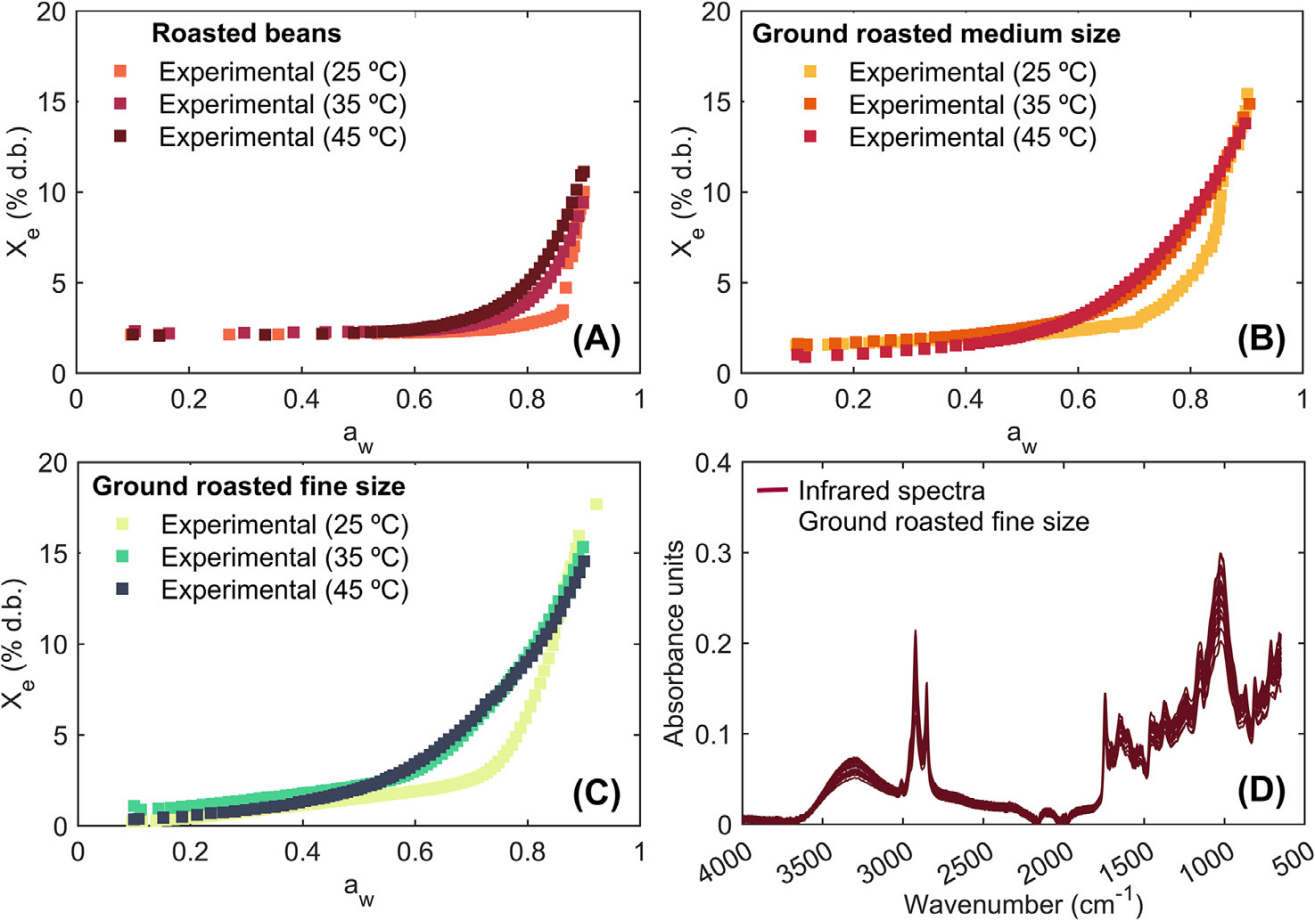
Example of the water sorption isotherms of roasted specialty coffee, whole beans (A), medium (B) and fine (C) ground roasted coffee at 25, 35 and 45 °C, and mid infrared spectra of fine ground roasted coffee.

**Fig. 3:**
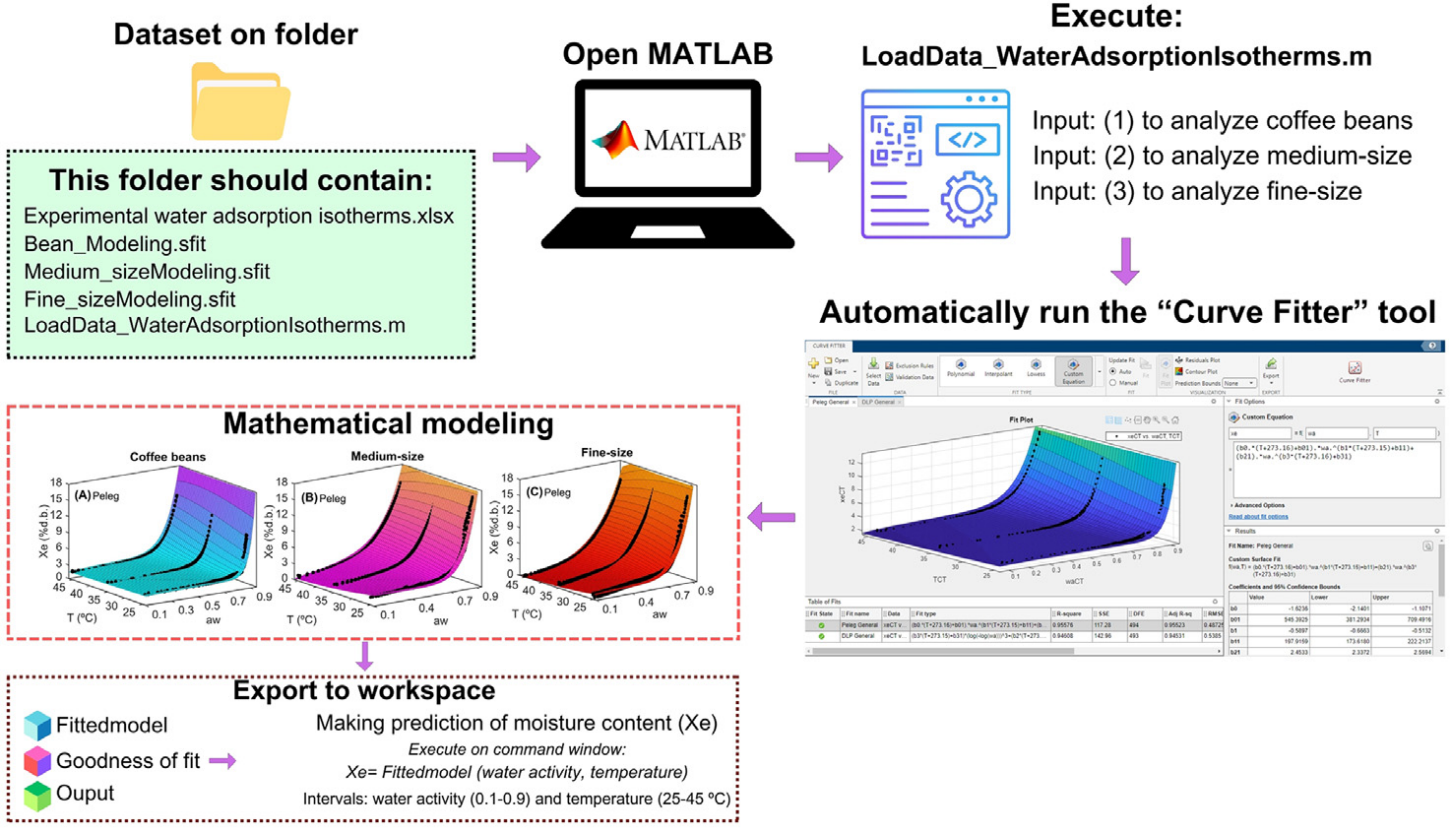
Assistance procedure for computer modeling of adsorption isotherms in roasted specialty coffee. A step-by-step interface designed to guide users in predicting the equilibrium moisture content of roasted coffee.

The experimental dataset was organized into four Excel files and four MATLAB files, each of which is described in detail below. They are the roasting curves applied to obtain a roast medium degree, the initial characterization of moisture content and water activity of roasted coffee samples, the infrared spectra of the ground product, and the adsorption isotherms of specialty coffee in whole beans, medium, and fine particle sizes detailed according to their experimental conditions and replicates. Additionally, the MATLAB files compile the statistical methodology followed to calibrate the statistical models on the adsorption isotherms. These data are presented in Fig. 1, Fig. 2, Fig. 3, and Table 1. Further, Fig. 3 illustrates the steps involved in the mathematical modeling procedure of the adsorption isotherms.

**Table 1:** Results of statistical model fitting for adsorption isotherms in roasted specialty coffee.

X_e_ (% d.b.)			
Type	Model	Parameters-CI (95 %)	Goodness of fit
Roasted coffee beans	Peleg	a_0_= –1.62 [–2.14, –1.11] a_01_= 545.40 [381.29, 709.50] a_1_= –0.59 [–0.66, –0.51] a_11_= 197.91 [173.62, 222.21] **a_2_= not significant** a_21_= 2.45 [2.33, 2.57] a_3_= 4.81×10^–3^ [8.02×10^–4^, 8.91×10^–3^] a_31_= –1.39 [–2.65, –0.14]	RMSE= 0.487 % d.b. R^2^_adj_= 95.52 %
	DLP	a_0_= –0.05 [–0.06, –0.03] a_01_= 17.05 [12.78, 21.32] a_1_= –0.02 [–0.04, –3.11×10^–4^] a_11_= 6.67 [1.24, 12.11] a_2_= 0.10 [0.07, 0.13] a_21_= –31.60 [–39.73, –23.46] a_3_= 0.03 [0.02, 0.04] a_31_= –9.22 [–12.51, –5.93]	RMSE= 0.54 % d.b. R^2^_adj_= 94.53 %

Medium ground coffee	Peleg	a_0_= –0.93 [–1.10, –0.82] a_01_= 317.02 [281.89, 352.15] a_1_= –0.16 [–0.18, –0.13] a_11_= 56.66 [47.28, 66.03] a_2_= 0.15 [0.11, 0.18] a_21_= –42.41 [–52.95, –31.84] a_3_= 0.06 [0.05, 0.07] a_31_= –17.84 [–20.87, –14.81]	RMSE= 0.53 % d.b. R^2^_adj_= 98.10 %
	DLP	a_0_= –0.04 [–0.05, –0.03] a_01_= 14.10 [11.23, 16.95] a_1_= –0.10 [–0.11, –0.09] a_11_= 30.01 [25.61, 34.41] a_2_= 0.13 [0.11, 0.15] a_21_= –38.95 [–45.90, –32.10] a_3_= 0.09 [0.07, 0.10] a_31_= –27.03 [–30.08, –24.10]	RMSE= 0.55 % d.b. R^2^_adj_= 97.83 %

Fine ground coffee	Peleg	a_0_= –1.01 [–1.08, –0.92] a_01_= 334.91 [310.18, 359.80] a_1_= –0.15 [–0.17, –0.12] a_11_= 51.97 [45.51, 58.42] a_2_= 0.26 [0.22, 0.29] a_21_= –75.29 [–86.21, –64.37] a_3_= 0.09 [0.08, 0.10] a_31_= –27.84 [–30.42, –25.26]	RMSE= 0.44 % d.b. R^2^_adj_= 98.70 %
	DLP	a_0_= –0.03 [–0.03, –0.02] a_01_= 9.36 [6.79, 11.92] a_1_= –0.09 [–0.10, –0.08] a_11_= 26.69 [22.58, 30.79] a_2_= 0.09 [0.07, 0.12] a_21_= –28.61 [–35.30, –21.91] a_3_= 0.07 [0.06, 0.08] a_31_= –21.92 [–24.86, –18.98]	RMSE= 0.58 % d.b. R^2^_adj_= 97.78 %

a_i_ (model parameters), DLP (Double Logarithm Polynomial), CI (confidence interval of model parameters), RMSE (root mean square error), R^2^_adj_ (adjusted coefficient of determination). Model parameters in bold were not statistically significant according to their 95 % CI estimation.

By employing the statistical procedure detailed below, the computational modeling of adsorption isotherms can be rigorously evaluated using a robust statistical foundation. This procedure standardizes the fitting of adsorption isotherms through advanced algorithms, improving modeling accuracy. In addition, it calculates various goodness-of-fit metrics and assesses the sensitivity of model parameters by determining their confidence intervals. These capabilities enable the identification of the most optimal sorption equation to effectively describe the coffee isotherms. Building upon this methodology, the procedure significantly improves accessibility to a robust statistical framework for adsorption isotherm modeling, making it suitable for users with varying degrees of expertise in computational modeling and software development. This advancement is particularly impactful in food science and food engineering, where precise adsorption isotherm modeling and the calculation of thermodynamic properties are essential for research and innovation.

**Roasting curves of specialty Coffee:** This dataset contains temperature profiles recorded during the coffee roasting process. The data are organized in a single table, with the first column representing processing time in seconds, while columns 2 through 10 provide temperature readings for each of the nine coffee samples analyzed. Temperature measurements were recorded at regular intervals (10 s) throughout the roasting process, offering a detailed view of temperature over time for each sample. The roasting process (Fig. 1) followed three distinct temperature phases. Initially, the roasting temperature was set to 178 ± 2 °C, after which the coffee samples were introduced into the roasting chamber. As the process began, the system temperature gradually decreased, reaching 125 ± 2 °C at 100 s, until the bean temperature equilibrated with the chamber temperature, marking the dehydration phase. Subsequently, temperatures remained stable between 100 and 220 s. Following this, the ascending phase, associated with coffee caramelization, led to a steady temperature increase, reaching 185 ± 2 °C by 550 s. The quality of the roasting process was monitored using the CieLab L* (lightness) coordinate, where an L* value between 18 and 25 indicates a medium roast degree [5]. L* measurements were performed using a spectrocolorimeter (CR-410, Konica Minolta, N.J., USA), yielding an average value of L*=24 ± 0.8, indicating a medium roast intensity.

**Initial moisture content and water activity:** This Excel file contains the initial characterization data for roasted specialty coffee. The initial two columns present the moisture content of whole roasted coffee beans, with measurements taken in quintuplicate and expressed as a percentage of moisture on both a wet and dry basis. The third column presents the water activity of the samples, which was measured in five replicates. These preliminary parameters furnish a fundamental comprehension of the coffee's hygroscopic properties, which are indispensable for assessing its shelf life.

**Experimental water adsorption isotherms:** This Excel file compiles adsorption isotherms for roasted specialty coffee, encompassing both whole beans and coffee ground to medium and fine particle sizes. The variables included in this dataset are water activity, which spans from 0.1 to 0.9, temperature at three distinct levels (25, 35, and 45 °C), and the equilibrium moisture content measured on both wet and dry bases. Fig. 2 illustrates examples of the adsorption isotherms for whole beans, as well as medium and fine ground coffee at varying experimental temperatures. Notably, the adsorption isotherms for whole beans (Fig. 2A) and ground coffee (Fig. 2B and C) display a J-curve shape, characteristic of type III isotherms as defined by the Brunauer-Emmett-Teller (BET) classification [8]. This pattern is typically observed in crystalline food matrices, such as baked and roasted products, and is indicative of food items with a high concentration of soluble components, particularly sugars [9].

**Infrared spectra of specialty Coffee:** This Excel file presents infrared spectra of roasted specialty coffee ground to a fine particle size. These spectra were recorded using fine size to facilitate the measurement of infrared spectra (as detailed in the EXPERIMENTAL DESIGN, MATERIALS AND METHODS section). The first column contains the wavenumber (cm^–1^) for the entire infrared spectrum, while columns 2 through 28 provide absorbance values for each sample across nine distinct coffee samples, with three replicates per sample (Fig. 2D). Furthermore, the last two columns (29 and 30) are the average spectra and standard deviation of all 27 spectra of samples. These spectra, collected from roasted coffee, offer valuable insights into the chemical composition and functional groups of roasted coffee.

**LoadData_WaterAdsorptionIsotherms:** This MATLAB file provides step-by-step instructions for loading an experimental dataset on the adsorption isotherms of roasted specialty coffee beans (including medium and fine ground coffee) and modeling them using both the Peleg and DLP models. Upon executing this file in the MATLAB editor, the program prompts the user to select the type of coffee for analysis. A user input of 1 runs the **Bean_Modeling.sfit** file, an input of 2 runs the **Medium_sizeModeling.sfit** file, and an input of 3 runs the **Fine_sizeModeling.sfit** file. In each of these “Curve Fitting” files, an optimization problem is solved with the objective of fitting the adsorption isotherms of roasted coffee beans. This optimization problem considers both the Peleg (Eq. (1)) and DLP ((2), (3)) models, with their respective parameters (a_i_) as decision variables, and uses the mean square error (MSE, Eq. (4)) as the objective function [10]. The goodness of fit of the models was assessed by root mean square error (RMSE, Eq. (5)) and adjusted coefficient of determination (R^2^_adj_, (6), (7)).

(1) $$X_{e}=b_{0}{a_{w}}^{b_{1}}+b_{2}{a_{w}}^{b_{3}}$$

(2) $$X_{e}=b_{0}+b_{1}x+b_{2}x^{2}+b_{3}x^{3}$$

(3) $$x=\text{ln}(-\ln a_{w})$$

(4) $$\text{MSE}=\frac{\sum_{i=1}^{N}{\left(X_{e}-\;X_{\text{epred}}\right)}^{2}}{N}$$

(5) $$\text{RMSE}=\sqrt{\text{MSE}}$$

(6) $$R^{2}\left(\%\right)=100-\frac{\sum_{i=1}^{N}{\left(X_{e}-\;X_{\text{epred}}\right)}^{2}}{\sum_{i=1}^{N}{\left(\bar{X_{e}}-\;X_{\text{epred}}\right)}^{2}}$$

(7) $$R_{\text{adj}}^{2}\left(\%\right)=100-\left(\frac{N-1}{N-M}\right)\left(100-R^{2}\right)$$

Where X_e_ and X_epred_ are the experimental and predicted equilibrium moisture content values (% dry basis, d.b.), a_w_ is the water activity, $\bar{X_{e}}$ is the mean of X_e_, R^2^ is the coefficient of determination, N is the number of data points, and M is the number of model's parameters. These metrics were adequate to define if a calibrated model can be considered as reasonable for practical applications in real scenarios (model with lower figures of RMSE and R^2^_adj_ >98 %) [11].

Fig. 3 illustrates the procedure for computer modeling of adsorption isotherms for roasted specialty coffee. A step-by-step interface was developed in MATLAB, guiding users through the application of calibrated Peleg and DLP models for the adsorption isotherms of whole coffee beans and medium/fine ground coffee. To utilize these calibrated statistical models, users must have MATLAB installed (version 2020a or later). Additionally, the files displayed in Fig. 3 should be available in the user's current directory before opening MATLAB. Once MATLAB is launched, users should run the script **LoadData_WaterAdsorptionIsotherms** and select one of the predefined options. This will trigger the “Curve Fitter” tool, which presents the interface where the Peleg and DLP models have been calibrated using the experimental adsorption isotherms. Within this interface, users could also integrate other models by creating a new window. In the custom equation section, both theoretical models (such as Guggenheim-Anderson-de Boer; GAB and BET) and empirical models (e.g., Oswin, Kuhn, Halsey, Henderson, Smith, among others) could be implemented and automatically adjusted using the adsorption isotherms of roasted specialty coffee.

Once a model is selected for prediction purposes, the MATLAB object must be saved as described in Fig. 3. By selecting the export option, the workspace will contain the “Fittedmodel”, “Goodness of fit” and “Output” objects, which are essential for predicting equilibrium moisture content in coffee as a function of water activity and temperature. To calculate the moisture content, users could execute the command *Fittedmodel(water activity value, temperature value)* in the command window. To avoid inaccuracies in predicting the equilibrium moisture content of coffee due to extrapolation beyond the established boundary conditions in the mathematical model, water activity and temperature values must be maintained within the range of 0.1 to 0.9 for water activity and 25 to 45 °C for temperature. The goodness of fit metrics and algorithmic details used in model fitting are stored in the “Goodness of fit” and “Output” objects, respectively.

**Fig. 4:**
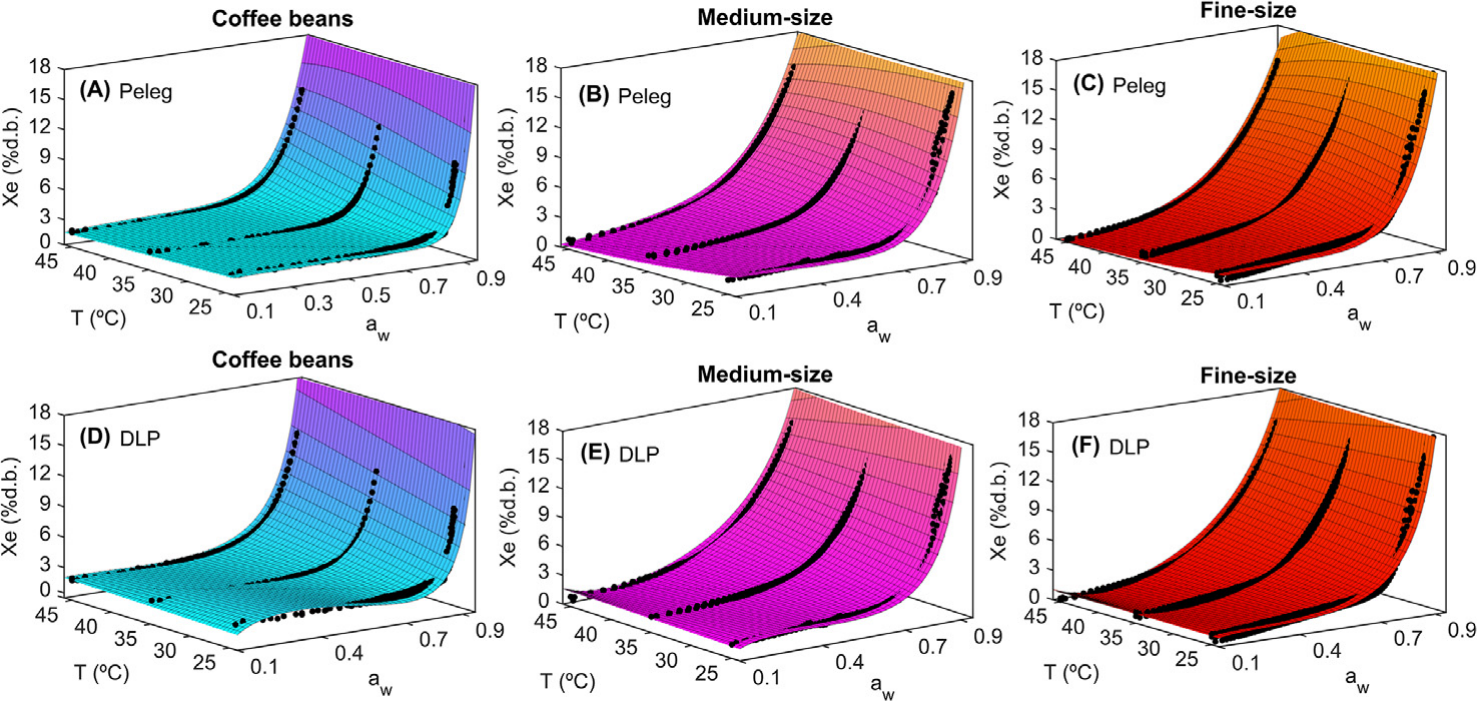
Response surface plots illustrating the adsorption isotherms of roasted specialty coffee modelled by the Peleg and DLP equations for whole beans (A, D), medium (B, E), and fine (C, F) ground roasted at 25, 35 and 45 °C.

The statistical results obtained from the mathematical modeling of the Peleg and DLP models are described in Table 1, while their capability to represent the influence of temperature and water activity on the equilibrium moisture content is depicted in Fig. 4 for whole beans (Fig. 4A and D), medium size (Fig. 4B and E) and fine size (Fig. 4C and F), respectively.

The statistical results of the adsorption isotherm fitting revealed RMRE values ranging from 0.44 to 0.58 % d.b. and R^2^_adj_ values between 94.53 % and 98.70 %, indicating high goodness of fit for the calibrated Peleg and DLP models in describing the influence of water activity and temperature on the equilibrium moisture content for whole beans, as well as medium and fine ground coffee (Table 1). Fig. 4 illustrates the experimental and modeled adsorption isotherms for whole coffee (Fig. 4A and D), medium ground (Fig. 4B and E), and fine ground coffee (Fig. 4C and F). Both the Peleg and DLP models successfully captured the adsorption isotherms, demonstrating their ability to accurately represent the type III adsorption curve, as well as the effect of temperature on equilibrium moisture content. The calibrated models, including their parameters and confidence intervals, could serve as virtual representations of roasted coffee bean storage and could be utilized to optimize both water activity and temperature, thereby maximizing moisture stability and preserving the quality of roasted specialty coffee.

This work represents the first dataset and statistical tools for computer-aided mathematical modeling of adsorption isotherms in coffee. Future research should focus on investigating the adsorption isotherms of roasted specialty coffee processed using different postharvest methods and degrees of roasting, while also considering additional process variables that may influence moisture stability and sensory quality attributes. Future research should also focus on developing advanced statistical procedures that enable the generalization of current sorption models, allowing for the integration of additional process variables (roasting degree, postharvest processing methods, among others) into the mathematical modeling. This would enhance the robustness and precision of predictions, making the mathematical tools more applicable and reliable for real applications in the coffee industry. Such advancements will improve the accuracy of sorption models and contribute to more effective decision-making and process optimization in industrial settings.

## 4 Experimental Design, Materials and Methods

**Fig. 5:**
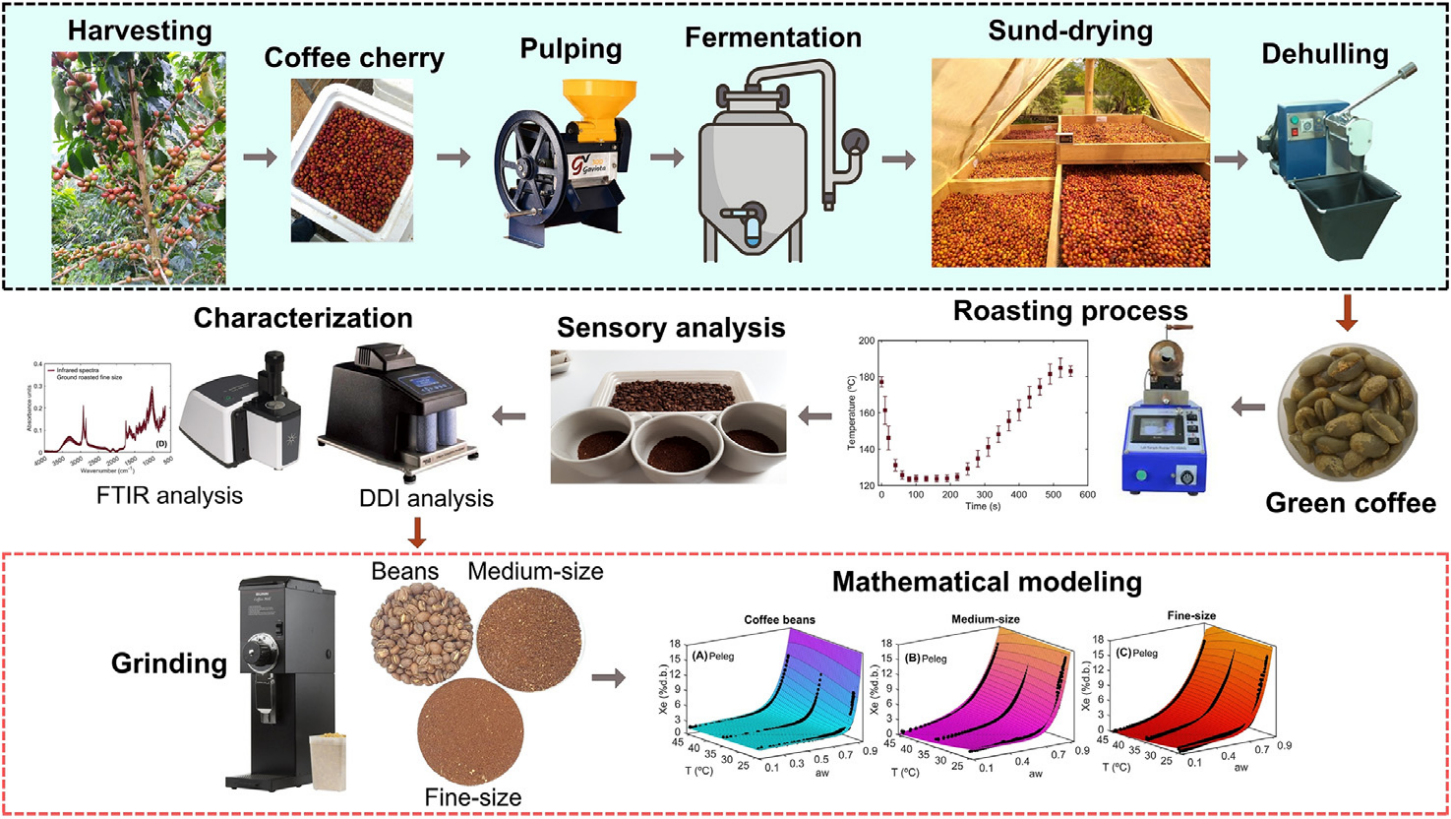
Flow chart illustrating the experimental procedure used to process coffee samples for obtaining the dataset and statistical tools.

The processing procedure illustrating the experimental methodology followed to process the coffee samples for obtaining the dataset and statistical tools is described in Fig. 5. Nine coffee cherry samples (10 kg each, *Coffea arabica* L. Colombian variety) were harvested from different growing areas in the Santa Maria, Huila Region of Colombia. Once the samples were collected, they were stored in refrigerated containers at 8 °C (Fig. 5) and transported for 4 h to be processed in the Centro Surcolombiano de Investigación en Café (CESURCAFÉ) in Neiva-Huila, Colombia. Firstly, the cherry beans were washed and the float beans were removed. Then, the samples were pulped using a pulping machine (Gaviota 300, Ingesec, Colombia). Coffee samples were processed via the wet postharvest method, which involves 18 h of fermentation in plastic containers [12], washing to remove the excess of coffee mucilage, and sun-drying to a moisture content ranging between 10 % and 12 % wet basis (% w.b.). Moisture content during sun-drying was monitored using a portable grain moisture meter (Kett PM-450, Science of Sensing, Japan). Sun-drying took place daily from 9 a.m. to 5 p.m. in conditions of 36 ± 3 °C and relative humidity between 20 % and 42 %. Subsequently, the dried parchment coffee beans were hulled using a hulling machine (ING-C-250, Ingesec, Colombia). Then, samples of 150 g of green coffee beans were roasted using rotary equipment (TC-150R, Quantik, Colombia), which allows for accurate control of time and temperature. The roasting process has already been detailed in the DATA DESCRIPTION section. Roasted coffee samples were sensory analyzed by a panel of five expert tasters, following the protocol outlined by the Specialty Coffee Association (SCA, 2020) and classified according to specialty coffee standards [13]. As the cup scores from the sensory analysis ranged from 84 to 88, the samples were considered to be high quality specialty coffees.

The initial characterization of the roasted samples entailed the determination of their moisture content, water activity, and infrared spectra. To achieve this, 5 g of each sample per triplicate was dried in an oven (UF55, Memmert GmbH + Co.KG, Schwabach, Germany) at 105 ± 1 °C until a constant weight was reached, which took approximately 24 h The results were expressed as a percentage on a wet and dry basis. The water activity was determined in triplicate using a vapor sorption analyzer (VSA Aqualab, Decagon Devices, Inc. Pullman, WA). Infrared spectra of the roasted samples were obtained using a Fourier-transform infrared (FTIR) spectrophotometer (Cary 630, Agilent Technologies, USA) equipped with a horizontal attenuated total reflectance (ATR) accessory comprising a diamond ATR and a ZnSe cell. The ATR-FTIR analysis was conducted in a controlled environment with temperature of 25 ± 0.5 °C [14]. The roasted coffee beans were ground using a Bezzera grinder (BB004NR0IL2, Italy), then sieved to isolate particles with of 250 µm diameter. Approximately 1 g of ground coffee was placed on the ATR accessory and compressed. Prior to each measurement of a sample, background readings were taken. The spectral data were recorded across a wavenumber range of 4000 to 650 cm^−1^, with a resolution of 8 cm^−1^ and a scan rate of 20. A background correction was applied [5]. The adsorption isotherms were determined through the use of the dynamic dewpoint isotherm (DDI) technique, employing a vapor sorption analyzer (VSA Aqualab, Decagon Devices, Inc. Pullman, WA). All experiments were conducted in triplicate at a range of water activity between 0.1 and 0.9, with 0.01 water activity intervals, at three different temperatures: The temperatures used were 25 °C, 35 °C, and 45 °C. In order to obtain the isotherms, a constant airflow of 100 mL/min was considered. In each test, 3.5 g of roasted coffee beans and ground coffee samples (both medium and fine particle sizes) were placed inside the DDI instrument. Subsequently, the adsorption isotherms were mathematically modelled using the procedure detailed in the DATA DESCRIPTION section.

## Limitations

None.

## Ethics Statement

The dataset acquired in this study did not involve human subjects, animal experiments, or data obtained from social media platforms.

## CRediT authorship contribution statement

**Gentil A. Collazos-Escobar:** Conceptualization, Methodology, Software, Data curation, Visualization, Writing – original draft. **Andrés F. Bahamón-Monje:** Software, Data curation, Writing – original draft. **Nelson Gutiérrez-Guzmán:** Supervision, Writing – review & editing.

## Data Availability

Mendeley DataStatistical dataset on water sorption isotherms in roasted specialty coffee (Coffee arabica L.) (Original data).
